# Graduated exposure to traumatic events decreases distress during prolonged exposure therapy for posttraumatic stress disorder

**DOI:** 10.1007/s10865-026-00666-2

**Published:** 2026-05-24

**Authors:** Kiara H. Buccellato, Hailey N. Brown, William A. Price, Casey L. Straud, Tabatha H. Blount, Edna B. Foa, Lily A. Brown, Carmen P. McLean, Richard P. Schobitz, Bryann B. DeBeer, Joseph Mignogna, Brooke A. Fina, Wyatt R. Evans, Brittany N. Hall-Clark, Timothy O. Rentz, Christian C. Schrader, Jeffrey S. Yarvis, Hunter Hansen, Vanessa M. Jacoby, Jose Lara-Ruiz, Willie J. Hale, Lauren M. Koch, Kelsi M. Gerwell, Stacey Young-McCaughan, Brett T. Litz, Eric C. Meyer, Douglas E. Williamson, John D. Roache, Allah-Fard M. Sharrieff, Barbara L. Niles, Terence M. Keane, Alan L. Peterson

**Affiliations:** 1 Department of Psychology, College for Health, Community and Policy, The University of Texas at San Antonio, 1 UTSA Circle, San Antonio, TX 78249 USA; 2 Department of Psychiatry and Behavioral Sciences, Joe R. & Teresa Lozano Long School of Medicine, The University of Texas at San Antonio, 7703 Floyd Curl Dr., MC 7747, San Antonio, TX 78229 USA; 3 Research and Development Service, South Texas Veterans Health Care System, 7400 Merton Minter, San Antonio, TX 78229 USA; 4 Department of Psychiatry, Center for the Treatment and Study of Anxiety, Perelman School of Medicine, University of Pennsylvania, 3535 Market St Suite 601N, Philadelphia, PA 19104 USA; 5 Dissemination and Training Division, National Center for PTSD, VA Palo Alto Health Care System, 795 Willow Rd, Menlo Park, CA 94025 USA; 6 Department of Psychiatry and Behavioral Sciences, Stanford University School of Medicine, 291 Campus Drive, Stanford, CA 94305 USA; 7 Department of Behavioral Health, Brooke Army Medical Center, Joint Base San Antonio-Fort Sam Houston, 3551 Roger Brooke Dr, Fort Sam Houston, TX 78234 USA; 8 Commissioned Corps Headquarters, U.S. Public Health Service, 1101 Wootton Pkwy, Rockville, MD 20852 USA; 9 VISN-17 Center of Excellence for Returning War Veterans, Central Texas Veterans Health Care System, 4800 Memorial Dr Bldg 93, Waco, TX 76711 USA; 10 Department of Veterans Affairs, Rocky Mountain Mental Illness Research Education and Clinical Center for Suicide Prevention, 1700 N Wheeling St, Building A2, Aurora, CO 80045 USA; 11 Department of Physical Medicine and Rehabilitation, University of Colorado, 12631 E 17th Ave, Aurora, CO 80045 USA; 12 Department of Behavioral Health, Carl R. Darnall Army Medical Center, 6065 Santa Fe Ave, Fort Hood, TX 76544 USA; 13 School of Social Work, Tulane University, 127 Elk Pl, New Orleans, LA 70112 USA; 14 Department of Psychiatry, Boston University Chobanian & Avedisian School of Medicine, 801 Massachusetts Ave, 1st Floor, Boston, MA 02118 USA; 15 Massachusetts Veterans Epidemiology Research and Information Center, VA Boston Healthcare System, 150 S Huntington Ave, Jamaica Plain, MA 02130 USA; 16 Department of Counseling and Behavioral Health, University of Pittsburgh, 119 University Pl, Pittsburgh, PA 15213 USA; 17 Department of Psychiatry and Behavioral Sciences, Duke University School of Medicine, 905 W Main St, Durham, NC 27701 USA; 18 Durham VA Health Care System, 508 Fulton St, Durham, NC 27705 USA; 19 Department of Homeland Security, 11226 NW 20th St, Miami, FL 33172 USA; 20 Behavioral Science Division, National Center for PTSD, VA Boston Healthcare System, 150 South Huntington Avenue, Boston, MA 02130 USA

**Keywords:** Prolonged exposure therapy, Imaginal exposure, Posttraumatic stress disorder, Subjective units of distress, Graduated exposure

## Abstract

Prolonged exposure (PE) is an evidence-based treatment for PTSD. However, the imaginal exposure portion of PE can be distressing for patients, and despite many service members reporting multiple traumatic events, traditional PE focuses solely on the most distressing event. This article evaluates a modified version of PE that considers the need for distress-reduction in trauma-focused treatments. We compared distress during two methods of imaginal exposure: (1) standard exposure, where participants focused on their most distressing event; and (2) graduated exposure, where participants focused on their top three most distressing events, from third most distressing to most distressing. Mean peak subjective units of distress scores (SUDS) were compared across groups. Participants were 199 active duty personnel and veterans (79.9% men; mean age 38.5 years). There was a significant overall effect of group on peak SUDS, *F*(3,359) = 12.46, *p* < .001. Participants receiving standard exposure reported an average peak SUDS of 88.1/100 (*SD* = 13.4), compared to 75.2 (*SD* = 22.5) for graduated imaginal exposure participant’s most distressing event. Participants in the graduated group also reported significantly lower peak SUDS for their second (M = 72.23, SD = 20.6; t(183) = 5.49, *p* < .001, d = .81) and third most distressing events (M = 74.63, SD = 21.1; t(183) = 466, *p* < .001, d = .69) compared to mean SUDS rating for the standard exposure group. Incorporating a graduated approach appears to mitigate average peak distress from participating in the imaginal exposure component of PE.

*Trial registration* These data were collected as part of a larger, randomized clinical trial that was registered with ClinicalTrials.gov (Identifier NCT03529435).

Prolonged exposure therapy (PE; Foa et al., 2019) is an evidence-based, trauma-focused, cognitive behavioral therapy for posttraumatic stress disorder (PTSD) recommended by leading clinical practice guidelines (American Psychological Association, 2025; Forbes et al., 2020; Forman-Hoffman et al., 2018; U.S. Department of Veterans Affairs & U.S. Department of Defense [VA/DoD], 2023). PE typically consists of 8–15 treatment sessions and begins with patients identifying their most distressing traumatic experience (i.e., the index event). Over the course of treatment, patients use imaginal exposure during psychotherapy sessions to revisit memories of and process emotions and cognitions related to their target trauma. In addition, patients complete behavioral exposure as homework to gradually approach objectively safe reminders of the traumatic event in real-life situations (Foa et al., 2019).

Despite the effectiveness of PE, trauma-focused treatments such as PE are associated with higher dropout rates compared to non-trauma-focused interventions, such as Present Centered Therapy (Imel et al., 2013; Lewis et al., 2020). Although emotional activation is considered a necessary component of successful PE treatment (Foa & Kozak, 1986; Foa et al., 2019), it is also a commonly cited explanation for high dropout rates (Hundt et al., 2020; Wells et al., 2023). For example, a study by Hundt et al. (2020) found that 60% of the participants who dropped out said it was because the treatment was too stressful.

Most behavioral treatments for anxiety disorders involve the development of a hierarchy of anxiety-provoking stimuli followed by graduated exposure to these stimuli starting with less distressing stimuli and then progressing to stimuli higher on the hierarchy (e.g., Wolpe, 1990). This is the approach used with the between-sessions, behavioral-exposure portion of PE (i.e., the in vivo component), in which therapists assist patients in the development of a hierarchy of trauma triggers and reminders to be used during homework exercises to gradually approach more distressing stimuli. In contrast, the imaginal exposure portion of PE targets the memory of the most distressing trauma in the first exposure and as the main focus of treatment. The initial development of PE involved the treatment of women sexual assault survivors for whom there was often a clearly identified target trauma or index event that was the focus of imaginal exposure (Foa et al., 1991). When PE was subsequently evaluated for the treatment of combat-related PTSD in active duty military personnel (Foa et al., 2018), the identification of a single index event was difficult because of the multiple trauma exposures that are often experienced in a combat environment (Benfer et al., 2024; Presseau et al., 2019; Stein et al., 2012). As a result, the standard imaginal exposure approach was modified in a subsequent clinical trial to assess the top three most distressing traumas and to then address all three of the trauma memories beginning with the least distressing trauma and progressing to the most distressing trauma (Peterson et al., 2023).

Distress reduction over the course of treatment is correlated with lower posttreatment PTSD symptoms (e.g., Bluett et al., 2014); however, it is not clear if a minimum peak distress threshold must be attained to facilitate PTSD treatment gains. In an effort to further improve PE treatment outcomes, modifications to traditional PE have aimed to keep peak distress lower, with the goal of improving treatment retention without negatively impacting outcomes. Addressing multiple events over the course of treatment, starting with an event that the patient deems relatively less distressing, may facilitate lower levels of patient distress during treatment. This in turn may facilitate increased mastery of the exercise and help the patient better tolerate exposure to more distressing trauma memories later during treatment. Such an approach may reduce dropout rates.

The aim of this study was to compare peak participant-reported distress levels across two PE delivery methods to determine if subjective distress differs when participants address (1) the single most distressing event versus (2) the top three most distressing events in order from least distressing to most distressing. The authors hypothesized that, compared to participants addressing only their single most distressing event, those addressing their top three most distressing events would demonstrate lower peak distress for each of their three events, as well as for all events combined when looking at peak distress across all three events in the graduated group.

## Methods

### Participants and procedure

These data were collected as part of a larger randomized clinical trial evaluating two methods of PE delivery that took place between 2017 and 2019 (see Peterson et al., 2018 and Peterson et al., 2023 for full trial details and primary outcomes). Active duty service members and veterans with PTSD (N = 234) from four study sites located in Texas were recruited to participate in the primary study. Participants were randomized to receive one of two PE treatment protocols: Massed PE (M-PE) or Intensive Outpatient Program PE (IOP-PE).

#### Massed prolonged exposure

M-PE consisted of 15 PE sessions of 90 min each over the course of 3 weeks. It included an overview of the treatment process and rationale, a trauma interview to identify the most distressing traumatic event, development of an in vivo trauma hierarchy to be used to guide homework assignments over the course of treatment, and imaginal exposure to the index event identified as the most distressing event, as is consistent with traditional PE treatment, starting in session 3 (Foa et al., 2019). M-PE has been shown to be non-inferior to traditional weekly/bi-weekly PE spaced over the course of approximately 8 weeks for reducing PTSD symptoms in the active duty military population (Dell et al., 2023; Foa et al., 2018). Participants in this group were also provided Team-Based Treatment (detailed below) to maximize treatment completion.

#### Intensive outpatient program prolonged exposure

IOP-PE consisted of the same standard PE components as described in the M-PE section above, over the course of 15 90-min PE sessions for 3 weeks, but it also added eight hypothesized treatment enhancements:

1. *Identification of the top three traumas*. Participants worked with their clinical provider to identify their top three most distressing events. These events were rank ordered by the participant as “first most distressing event”, “second most distressing event” and “third most distressing event”.
2. *Graduated imaginal exposure*. Starting with treatment session 3, the clinical provider led participants through imaginal exposure encounters during which participants were asked to provide detailed recounting of their third most distressing event in a safe, controlled environment. Participants repeated this exercise at all subsequent treatment sessions, until imaginal exposure to their third most distressing event no longer elicited strong feelings of distress. Participants then addressed their second most distressing event using imaginal exposure, followed finally by their first most distressing event.
3. *Team-based treatment*. All participants were assigned to a primary therapist who spearheaded their treatment team. In addition, each treatment team included a secondary therapist and clinical provider extenders, who assisted with completion of treatment exercises. This method aimed to mitigate missed sessions by ensuring provider availability over the course of treatment.
4. *Clinic-based completion of homework*. Daily homework assignments, including iv vivo exposures and listening to audio recordings of imaginal exposure sessions, were completed in clinic by IOP-PE participants. This modification strove to minimize participant avoidance behaviors outside of formal PE treatment sessions and optimize homework completion between sessions.
5. *Brief therapist feedback sessions*. Participants completed two brief feedback sessions with a member of their treatment team each day. These sessions occurred after completion of daily in vivo exposure homework and after listening to the audio recordings of their imaginal exposure sessions. These feedback sessions allowed participants to receive emotional support and assistance in cognitive processing after completing their homework assignments.
6. *Enhanced social support*. A social-support person, such as a spouse or close friend, was included in treatment session 2, during which the clinical provider detailed the treatment rationale, common reactions to trauma and treatment, and the purpose of homework assignments. The goal of this modification was to mitigate potential encouragement of avoidance behaviors sometimes inadvertently exhibited by patient’s social support systems.
7. *Brief timeline review of all traumas*. After completing imaginal exposure for the top three reported events, participants briefly reviewed all lifetime traumatic events. The goal of this brief timeline review was to demonstrate that the skills the participant acquired through addressing their top three traumatic events could be utilized to address other distressing experiences after treatment completion.
8. *Posttreatment booster sessions*. Participants completed 3, 1-h booster sessions 1, 3, and 7 weeks posttreatment to allow for a more tapered discontinuation of treatment and assistance in transitioning the skills learned during treatment to their daily life environment long-term.

Primary outcomes included clinician-rated (Clinical-Administered PTSD Scale for DSM-5 [CAPS-5]; Weathers et al., 2018) and self-report (PTSD Checklist for DSM-5 [PLC-5]; Weathers et al., 2013) measures of PTSD symptom reduction from pretreatment to three posttreatment follow-up timepoints (1-month, 3-months, and 6-months posttreatment). PTSD diagnostic remission rates at each posttreatment timepoint were evaluated as secondary outcomes. The primary analyses of this trial revealed significant reductions in clinician-rated and self-report PTSD severity via the CAPS-5 and PCL-5, respectively, from pre- to posttreatment in both treatment groups. Both groups maintained self-reported PTSD symptom reductions at 6-month follow-up, however, only the IOP-PE group maintained similar levels of clinician-rated treatment gains at 6-month follow-up compared to posttreatment (Peterson et al., 2023). PTSD diagnostic remission rates were 48% for IOP-PE participants and 61% for M-PE participants at posttreatment, and ~ 50% for both groups at 6-month follow-up (Peterson et al., 2023).

This secondary data analysis seeks to expand upon these primary findings by evaluating participant-reported feelings of distress across M-PE and IOP-PE to elucidate if between-group differences exist. Basis for these analyses is rooted in the theoretical rationale driving the first two augmentations of IOP-PE (described above), “Identification of the Top Three Traumas” and “Graduated Exposure”. Specifically, the researchers implemented the hierarchical approach to confronting distressing stimuli utilized in anxiety disorder treatments in the IOP-PE group by beginning with the participant’s third most distressing event and working-up to their most distressing event over the course of treatment in an attempt to mitigate severe emotional distress during treatment (Wolpe, 1990).

With this secondary analysis, the researchers aimed to evaluate how beginning imaginal exposure with the third most distressing event and working up to the second most distressing event, then the most distressing event over the course of treatment impacted participant’s subjective experience of emotional distress during treatment. The current analysis sought to compare the subjective distress experienced during imaginal exposure of the two treatment groups to determine the effect of graduated exposure (i.e., the IOP-PE group) vs. the standard exposure approach (from the M-PE group).

### Measures

#### Demographics and military characteristics

Participants completed the Demographics and Military Service Characteristics Form prior to start of treatment. This self-report measure included items addressing standard demographic characteristics, such as age, gender, race, and marital status, as well as military-related characteristics, such as branch, military pay grade, and years of service in the military.

#### Subjective units of distress scale

Participant-reported levels of distress were collected during imaginal exposure portion of PE during sessions 3–15 using the Subjective Units of Distress Scale (SUDS; Wolpe, 1969). Peak SUDS were defined as the highest distress rating endorsed by participants for each of their traumatic events, with 0 indicating “State of Absolute Calmness” and 100 indicating “Worst Anxiety Ever Experienced.” Average peak SUDS were calculated for the most distressing event (standard imaginal exposure group); the most, second most, and least distressing event (graduated imaginal exposure group); and all events combined (graduated imaginal exposure group). The team then identified the maximum distress reported by participants for each event and overall. Five participants requested to report their SUDS ratings on a scale from 0 to 10 (Wolpe, 1990). These scores were transformed to a 0 through 100 scale by multiplying their ratings by 10, thus allowing them to be included in these analyses.

### Data analysis

Analyses were conducted using SPSS statistical software version 29 (IBM). ANOVA with planned contrasts was used to investigate pairwise differences in average peak SUDS scores between the most distressing event during standard imaginal exposure and the most distressing event during graduated imaginal exposure. Planned contrasts compared the most distressing event during standard imaginal exposure to the second most distressing event, least distressing event, and the average of all events combined for the graduated imaginal exposure group. Cohen’s d was calculated to further explicate the nature of significant effects.

## Results

### Sample

Data analyzed were collected from participants (N = 199) seeking treatment for PTSD at three of the four original study performance sites. SUDS scores were unavailable for one study site (n = 35 of the original study sample N = 234). The sample was mostly male (79.9%; 159/199) with a mean age of 38.5 years old (SD = 7.4). The sample was enthoracially diverse, with 41.2% (82/199) identifying as Non-Hispanic White, 25.6% (51/199) Hispanic, 28.6% (57/199) African American, and 4.5% (9/199) other. See Table 1 for full demographic and military characteristic information. Chi-square tests of independence for categorical variables and independent samples t-tests for continuous variables were run to evaluate if demographic and military characteristics varied between groups. No significant differences were found between the treatment groups for age, gender, rank, race & ethnicity, education, military status, branch, highest rank, months in military, or years in military. Marital status differed between the treatment groups, with a larger portion of IOP-PE participants married/living with partner (χ^2^(2) = 6.14, *p* = .046).

**Table 1:** Demographic and military characteristics

	Participants, No. (%)		
Characteristics	Total (*N* = 199)	Graduated (*n* = 102)	Standard (*n* = 97)
Age, mean (*SD*), y	38.5 (7.4)	39.14 (7.0)	37.8 (7.8)
Gender			
Woman	40 (20)	17 (17)	23 (24)
Man	159 (80)	85 (83)	74 (76)
Marital Status			
Married/Living with partner	147 (74)	83 (81)	64 (66)
Single/Not living with partner	21 (11)	8 (8)	13 (13)
Divorced/separated	31 (16)	11 (11)	20 (21)
Race and ethnicity			
African American	57 (29)	30 (30)	27 (28)
Hispanic	51 (26)	28 (28)	23 (24)
Non-Hispanic White	82 (41)	39 (38)	43 (44)
Other	9 (5)	5 (5)	4 (4)
Education			
≤ Associates degree	136 (68)	72 (71)	64 (66)
4-year college degree	41 (21)	20 (20)	21 (22)
Graduate degree	22 (11)	10 (10)	12 (12)
Military status			
Active Duty	131 (66)	70 (69)	61 (63)
National Guard	2 (1)	0 (0)	2 (2)
Reserve	3 (2)	0 (0)	3 (3)
Retired	30 (15)	13 (13)	17 (18)
Veteran	33 (17)	19 (19)	14 (14)
Branch			
Army	164 (82)	84 (82)	80 (83)
Air Force	21 (11)	10 (10)	11 (11)
Navy	8 (4)	5 (5)	3 (3)
Coast Guard	2 (1)	1 (1)	1 (1)
Marines	4 (2)	2 (2)	2 (2)
Time in military, mean (*SD*), y	15.2 (6.8)	15.6 (6.5)	14.9 (7.1)
Military Pay Grade			
E-4 to E-6	115 (58)	57 (56)	58 (60)
E-7 to E-9	56 (28)	31 (31)	25 (26)
W-01 to O-6	28 (14)	14 (14)	14 (14)

E-4 to E-6 = junior noncommissioned officers; E-7 to E-9 = senior noncommissioned officers; W = warrant officer; O = officer

Available data were from three of the four study sites (N = 199) that recorded SUDS ratings. From the available data, SUDS scores were missing for n = 13 IOP-PE graduated imaginal exposure participants who either: (1) did not complete at least three treatment sessions or (2) did not address their top three most distressing events, and n = 1 M-PE imaginal exposure participant who did not address their most distressing event in treatment. A total of n = 96 M-PE participants had their most distressing events documented and were included in these analyses. A total of n = 89 IOP-PE participants had a documented third, second and first most distressing event (267 unique events total) and were included in these analyses.

### Differences in participant-reported distress

**Fig. 1:**
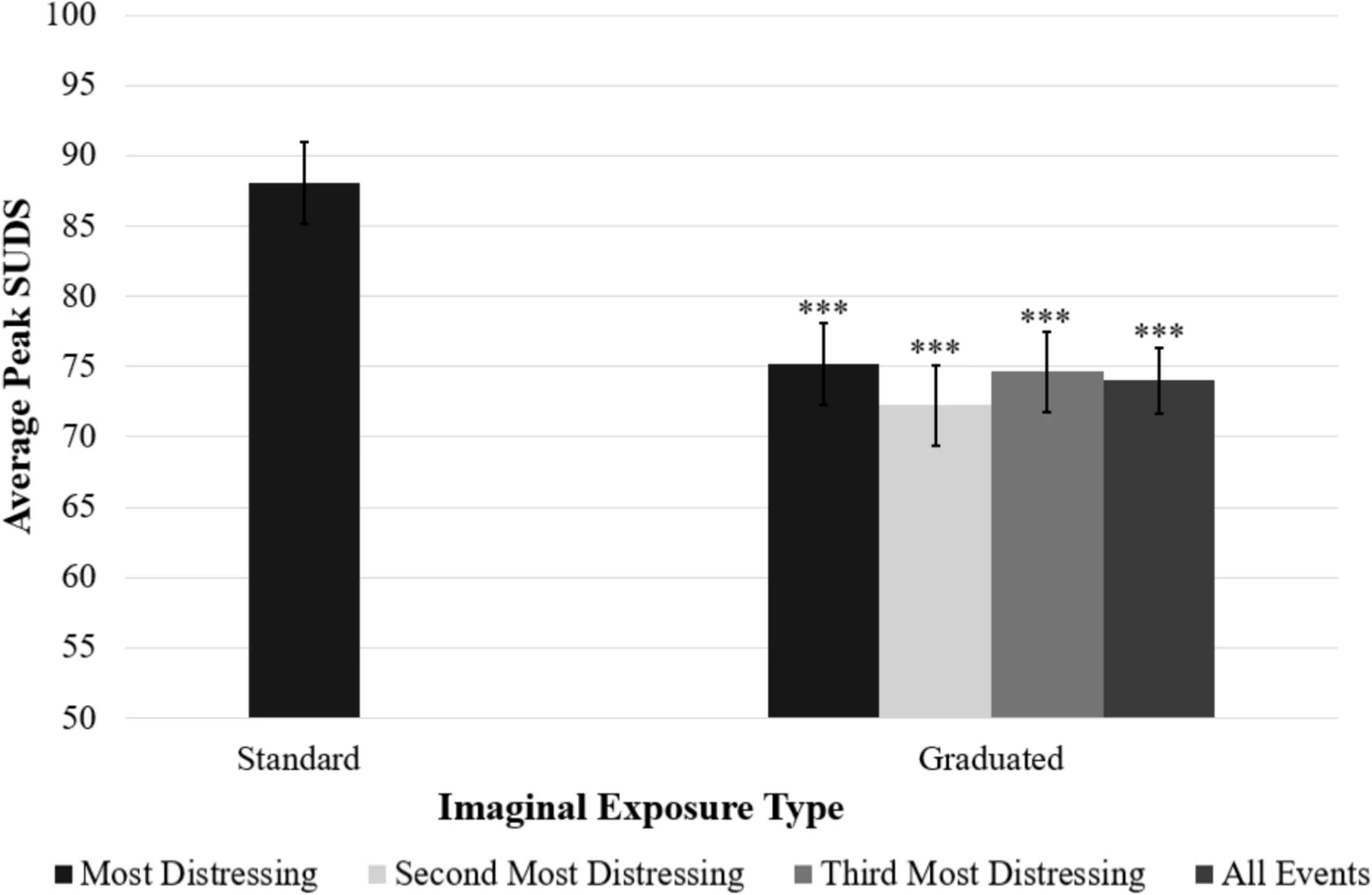
Average peak SUDS rating by group and event. *Note*. Average peak SUDS ratings are shown for standard imaginal exposure Most Distressing Event, graduated imaginal exposure top three most distressing events, and graduated imaginal exposure all events combined. Error bars show standard error. *** Denotes average peak SUDS rating is significantly different from standard imaginal exposure Most Distressing Average Peak SUDS rating at p < .001

There was a significant overall effect of treatment group on peak SUDS, F(3,359) = 12.46, p < .001. Planned contrasts revealed that the standard imaginal exposure group rated their most distressing trauma significantly higher with an average peak SUDS across all treatment sessions of 88.06 (*SD* = 13.39), as compared to 75.19 (*SD* = 22.52) for the most distressing trauma across all treatment sessions for participants using graduated imaginal exposure, *t*(183) = 4.46, *p* < .001, *d* = .70. Graduated imaginal exposure participants also reported significantly lower peak SUDS for their second most distressing event (M = 72.23, SD = 20.57) compared to standard imaginal exposure participants (M = 88.06, SD = 13.39) for their most distressing event, t(183) = 5.49, p < .001, d = .81. Similar results were found when comparing graduated imaginal exposure participant’s least distressing event (M = 74.63, SD = 21.08) compared to standard imaginal exposure participant’s most distressing event (M = 88.06, SD = 13.39; t(183) = 4.66, p < .001, d = .69). When comparing all events (i.e., standard imaginal exposure most distressing event and graduated imaginal exposure top three most distressing events) between groups, graduated imaginal exposure participants endorsed significantly lower average peak SUDS scores across all treatment sessions (*M* = 74.02, *SD* = 21.36) compared to standard imaginal exposure participants (M = 88.06, SD = 13.39; *t*(359) = 6.02, *p* < .001, *d* = .72; see Fig. 1).

## Discussion

This secondary data analysis of a clinical trial evaluating two methods of PE therapy in active duty service members and veterans sought to evaluate between-treatment group differences in participant-reported distress during imaginal exposure. The results demonstrated that a graduated approach to imaginal exposure, whereby participants address their third most distressing event at the start of treatment and work their way up to their second most distressing event, then finally their first most distressing event, produced lower levels of average peak distress across treatment sessions compared to addressing solely the participants most distressing event. These findings align with previous studies documenting the benefits of graduated exposure to anxiety- and fear-producing stimuli (Kaplan & Tolin, 2011).

In the context of anxiety disorder treatment, the two methods of imaginal exposure implemented here could be best conceptualized as flooding (M-PE group) and graduated exposure (IOP-PE group; Zoellner et al., 2008). Research has demonstrated the effectiveness of both methods for treating anxiety disorders, however a 2019 review of exposure therapy notes that the graduated, hierarchical method of addressing feared stimuli, starting with less distressing stimuli and proceeding to more distressing stimuli after habituation has occurred, is the prevalent clinical approach (Craske et al., 2012; Knowles & Olatunji, 2019). Despite this, a graduated approach has not been widely adopted nor studied for the imaginal exposure component of PTSD treatment. Apart from the main outcomes paper reporting the primary results of this clinical trial, the researchers were able to identify one other study that examined the clinical effects of graduated exposure therapy for PTSD (McLay et al., 2011; Peterson et al., 2023). McLay and colleagues (2011) examined the effects of virtual-reality graduated exposure therapy, versus broadly defined treatment-as-usual, on PTSD treatment outcomes in post-9/11 service members (McLay et al., 2011). They found that participants in the graduated group demonstrated significantly more PTSD symptom improvement than participants in the treatment-as-usual group, defined by number of participants per group who achieved a 30% or greater pre- to posttreatment improvement on the CAPS (McLay et al., 2011). Although this provides some support for the use of graduated exposure, it is limited by its use of a broad control group that does not directly compare graduated exposure to traditional exposure.

To the authors knowledge, the results presented herein are the first to compare participant-reported distress during the imaginal exposure portion of PE between graduated and traditional exposure groups. Exploring avenues for reducing patient distress during exposure therapy is critical, due to the existing perception of exposure therapy as distressing by both patients and providers (Alpert et al., 2024; Hundt et al., 2020; Schumacher et al., 2015). Previous literature has documented veterans’ anxiety surrounding trauma-focused treatments stemming from not wanting to talk about their traumatic events, heightened distress after initiation of treatment, and difficulty developing trust with their clinician that is likely related to the high levels of distress they experience during treatment (Alpert et al., 2024; Hundt et al., 2020; Lu et al., 2016; Wells et al., 2023). A hierarchical approach to imaginal exposure, in which patients progress from less to more distressing events, may reduce anticipatory anxiety associated with imaginal exposure. In a national qualitative study comparing treatment completers and treatment non-completers among veterans undergoing trauma focused therapy for PTSD the researchers found themes surrounding how completers versus non-completers attributed meaning to their treatment distress (Kehle-Forbes et al., 2022). Specifically, completers interpreted their feelings of distress as part of the treatment process, while non-completers interpreted their feelings of distress as signs that their symptoms were worsening and treatment was not working for them (Kehle-Forbes et al., 2022). Addressing the most distressing event at the beginning of treatment, as is traditional for PE, may result in higher feelings of distress, as indicated in the present analyses, and participants feeling as though the treatment does not work for them within the first few sessions. Kehle-Forbes and colleagues (2022) found that the majority of participants (70%) who dropped out of treatment did so between sessions 3 and 6, indicating they would have completed between one and three imaginal exposure treatment sessions prior to dropout. By easing participants into treatment with one of their less distressing events first, participants who otherwise may have dropped out early due to feelings of distress may be able to build greater confidence and comfort in the treatment over time. Alternatively, participants who interpreted their heightened distress as part of the treatment process may interpret the lower distress associated with graduated exposure as treatment not working for them. These findings should also be considered in the context of emotional processing theory, which underpins PE and posits that activation of the fear structure is necessary for corrective learning to occur. From this perspective, some degree of distress during imaginal exposure is thought to be essential for therapeutic benefit and should be considered in the context of gradual exposure (Foa & Kozak, 1986). Further research is needed to elucidate participant perception of distress in each PE method as well as the potential relationship between distress, symptom reduction, and treatment attrition.

The present study suggests that participants with PTSD who underwent a traditional approach (most distressing trauma only) to imaginal exposure experienced a higher level of peak distress over the course of therapy than participants who underwent a graduated approach. Average peak SUDS ratings were significantly lower among participants receiving graduated imaginal exposure across each event and all events combined compared to participants receiving standard imaginal exposure, with medium to large effect sizes. These results provide preliminary support for the potential adoption of the hierarchical, graduated approach to exposure already utilized widely in treatment of anxiety disorders for treatment of PTSD to facilitate lower levels of peak distress among patients undergoing treatment.

## Limitations

This study analyzed data collected from a larger randomized clinical trial aimed at evaluating two methods of PE delivery for symptoms of PTSD in active duty service members and veterans (Peterson et al., 2023). Although addressing the top three most distressing events in the IOP-PE group versus the single most distressing event in the M-PE group was a notable difference between groups, it encompasses only two of the eight distinctions in PE delivery between groups. It is possible that one or more additional components of the PE delivery could explain the differences seen in average peak SUDS between groups (e.g., enhanced social support through participation of spouse or significant other). A detailed description of the PE augmentation methodology used in each group was published previously and an overview of these augmentations are provided above (see “Methods” Section; Peterson et al., 2018).

## Future directions

This research provides initial support for the approach of addressing the top three most distressing traumas in order from least distressing to most distressing, rather than focusing solely on the single most distressing event during PE treatment. Future research would benefit from evaluating subjective distress over time, between groups where treatment modality is held constant with the exception of addressing top three most distressing events versus single most distressing event. This would allow not only for a more controlled examination of the effects of graduated imaginal exposure on not only patient-reported distress, but also of dropout over the course of treatment to determine if this augmentation supports increased treatment completion. Lower drop-out rates have been demonstrated in both massed and IOP PE, compared to traditional PE delivered once per week (Levinson et al., 2022). In this trial, treatment success and completion rates were high in both groups, with no significant differences found between groups (Peterson et al., 2023). However, the specific effects of graduated versus traditional imaginal exposure on treatment outcomes are not able to be evaluated directly using these data, given that it was not the only between group difference in treatment methodology utilized in this study.

Finally, addressing the top three traumatic events in PE treatment may also provide clinical benefits in relation to different symptom clusters of PTSD. Different types of traumatic experiences are often observed to be more strongly associated with different PTSD symptom clusters (Kelley et al., 2009). For example, combat-related trauma frequently correlates with greater intrusive and arousal symptom cluster scores, while sexual trauma often correlates with greater avoidance and cognition/mood symptom cluster scores, and specifically posttraumatic amnesia (Guina et al., 2018). Service members often experience more than one traumatic event, and these events often encompass more than one category, such as combat trauma, childhood trauma, military sexual trauma, etc. (Reger et al., 2019). The cumulative impact of these experiences may not be fully addressed in treatments that focus solely on the single most distressing event. Focusing on the top three events may allow service members to address experiences encompassing both military and non-military related trauma. Future research would benefit from evaluating the impact of addressing multiple traumatic events on the improvement of PTSD symptom clusters.

## Conclusion

Distress associated with discussing traumatic events may be mitigated through augmentation of PE to include addressing the top three most distressing events using graduated exposure. Trauma-focused therapies, including PE, are both effective and strongly recommended treatments for active duty service members and veterans with PTSD. However, failure to initiate treatment and early dropout are persistent problems. It is important to explore ways to make treatment more tolerable for service members and veterans who drop out of treatment early or fail to initiate treatment entirely.

## Data Availability

The data from this study are maintained at The University of Texas at San Antonio in the STRONG STAR Repository. Requests for access to the data, the analysis code, and associated materials may be emailed to repository@strongstar.org.
